# Common Oncogenic Mutations Are Infrequent in Oral Squamous Cell Carcinoma of Asian Origin

**DOI:** 10.1371/journal.pone.0080229

**Published:** 2013-11-04

**Authors:** Sharifah Nurain Syed Zanaruddin, Pei San Yee, Seen Yii Hor, Yink Heay Kong, Wan Maria Nabillah Wan Abd Ghani, Wan Mahadzir Wan Mustafa, Rosnah Binti Zain, Stephen S. Prime, Zainal Ariff Abd Rahman, Sok-Ching Cheong

**Affiliations:** 1 Department of Oral & Maxillofacial Surgery, Faculty of Dentistry, University of Malaya, Kuala Lumpur, Malaysia; 2 Oral Cancer Research Team, Cancer Research Initiatives Foundation, 2nd Floor Outpatient Centre, Sime Darby Medical Centre, Selangor, Malaysia; 3 Oral Cancer Research and Coordinating Centre, University of Malaya, Kuala Lumpur, Malaysia; 4 Department of Oral & Maxillofacial Surgery, Hospital Kuala Lumpur, Kuala Lumpur, Malaysia; 5 Department of Oral Pathology, Oral Medicine and Periodontology, Faculty of Dentistry, University of Malaya, Kuala Lumpur, Malaysia; 6 Centre for Clinical and Diagnostic Oral Sciences, Institute of Dentistry, Barts and The London School of Medicine and Dentistry, Queen Mary University of London, London, United Kingdom; Virginia Commonwealth University, United States of America

## Abstract

**Objectives:**

The frequency of common oncogenic mutations and *TP53* was determined in Asian oral squamous cell carcinoma (OSCC).

**Materials and Methods:**

The OncoCarta^™^ panel v1.0 assay was used to characterize oncogenic mutations. In addition, exons 4-11 of the *TP53* gene were sequenced. Statistical analyses were conducted to identify associations between mutations and selected clinico-pathological characteristics and risk habits.

**Results:**

Oncogenic mutations were detected in *PIK3CA* (5.7%) and *HRAS* (2.4%). Mutations in *TP53* were observed in 27.7% (31/112) of the OSCC specimens. Oncogenic mutations were found more frequently in non-smokers (*p* = 0.049) and *TP53* truncating mutations were more common in patients with no risk habits (*p* = 0.019). Patients with mutations had worse overall survival compared to those with absence of mutations; and patients who harbored DNA binding domain (DBD) and L2/L3/LSH mutations showed a worse survival probability compared to those patients with wild type *TP53*. The majority of the oncogenic and *TP53* mutations were G:C > A:T and A:T > G:C base transitions, regardless of the different risk habits.

**Conclusion:**

Hotspot oncogenic mutations which are frequently present in common solid tumors are exceedingly rare in OSCC. Despite differences in risk habit exposure, the mutation frequency of *PIK3CA* and *HRAS* in Asian OSCC were similar to that reported in OSCC among Caucasians, whereas *TP53* mutations rates were significantly lower. The lack of actionable hotspot mutations argue strongly for the need to comprehensively characterize gene mutations associated with OSCC for the development of new diagnostic and therapeutic tools.

## Introduction

Oral squamous cell carcinoma (OSCC), a subset of head and neck squamous cell carcinoma (HNSCC), is one of the most common malignancies with more than 400,000 of new cases diagnosed annually worldwide [1]. Particularly in South East Asia, the disease is reaching epidemic proportions with age-standardized rates (ASR) of 6.7 compared to 4.3 and 4.0 in Europe and America respectively [2]. The disease has significant physical and psychological morbidity and a survival rate of approximately 50% over 5 years, a figure that reflects the stage of the tumour at presentation and the development of loco-regional recurrences, distant metastases and second primary tumours. Survival rates have not improved for decades and taken together, the findings argue strongly for the need to develop new therapeutic strategies.

Cancer occurs due to the progressive accumulation of abnormalities in cellular DNA which, in turn, provide a selective growth advantage to cancer cells and facilitate metastatic dissemination [3]. Dysregulation of certain signaling pathways, together with chromosomal abnormalities, have been identified in HNSCC [4] and more recently, *TP53, CDKN2A, PIK3CA, PTEN* and *HRAS*, together with *FBXW7, NOTCH1, IRF6* and *TP63*, have been shown to play fundamental roles in the pathogenesis of HNSCC [5-7]. Further, the nature of gene mutation is thought to reflect the exposure to specific risk factors, with G > T transversions at non-CpG sites being characteristic of tobacco exposure [6,8]. However, these and other studies [5,9,10] have been undertaken using tissue specimens and cell lines from Caucasian populations where smoking and excessive alcohol consumption are primary risk factors. By contrast, very little is known about the spectrum of gene mutations in OSCC of Asian origin where the disease is most prevalent [1] and where the practice of betel quid chewing, with or without smoking has been demonstrated to be associated with the increase risk to oral cancer in about 50% of the patients [11-13]. 

Mutations in genes that play fundamental roles in driving cancer development have defined treatment protocols in a diverse group of tumor types [14,15], but information regarding oral squamous cell carcinoma is limited. In the present study, we used high-throughput mutational profiling to determine the prevalence of mutations at 238 sites across 19 oncogenes in Asian OSCC as well as *TP53* in 107 tissues and 16 cell lines. We demonstrate lower levels of *TP53* mutations but similar mutational frequencies in *HRAS* and *PIK3CA* in Asian OSCC compared to Caucasian OSCC. Most notably, we show that mutations in the 19 oncogenes are exceedingly low compared to other solid cancers including lung cancer where the etiological factors are similar to that of OSCC. The findings suggest that mutations other than those commonly seen in solid cancers may play an important role in driving OSCC and argue strongly for further comprehensive analysis of gene mutations in this tumor type. 

## Materials and Methods

### Ethics Statement

All of the clinical samples were obtained from patients with written informed consent, and this study was approved by the Institutional Review Board of the Faculty of Dentistry, University of Malaya (Medical Ethics Number: DF OS1002/0008/L). 

The 16 cell lines that were used in this study were established in our laboratory and have been described previously [16]. These were established from tissues that were collected with written informed consent and were approved by the Institutional Review Board of the Faculty of Dentistry, University of Malaya (Medical Ethics Number: DP OP0306/0018/L).

### Clinical samples and cell lines

 One hundred and thirty genomic DNA (gDNA) samples from 107 fresh frozen OSCC tissues, 16 oral squamous cell carcinoma (OSCC) cell lines and 7 control cell lines positive for specific mutations were included in this study. gDNA from OSCC tissues that had a minimum of 70% tumor coverage and the data associated with these specimens were obtained from the Malaysian Oral Cancer Database & Tissue Bank System (MOCDTBS) [17]. Information pertaining to the tissue specimens is shown in Table 1. Sixteen OSCC cell lines (Table S1 in File S1) were established from primary explant cultures in our laboratory, as described previously [16]. With the exception of ORL-156, all of the cell lines have been authenticated to tissues and/or blood samples. ORL-156 has a suspicious identity with a 60% match to the original tumor tissue. gDNA from seven cell lines which contained mutations in specific genes were kind gifts from Dr. Ramsi Haddad, Laboratory of Translational Oncogenomics, Karmanos Cancer Institute, Wayne State University, USA (Table S2 in File S1). Five of these lines originated from breast carcinomas [18,19], one was from an ovarian cancer [20] and another was from an ovarian cancer mouse xenograft. All gDNA extraction was performed using the QIAamp DNA mini kit (Qiagen, Germany), according to manufacturer’s recommendation and the quantity and quality of gDNA was determined using the NanoDrop ND1000 Spectrophotometer and gel agarose electrophoresis. 

**Table 1:** Demographics and clinico-pathological characteristics of patients included in the study.

**Variable**			**n=107**	**%**
Gender	Male		43	40.2
	Female		63	58.9
	Information unavailable		1	0.9
Age	Mean	58	--	--
	Range	58	--	--
Risk Habits	Exclusively smokers		12	11.2
	Exclusively betel quid chewers		35	32.7
	Exclusively alcohol drinkers		3	2.8
	*Two Habits*			
	Chewing + Smoking		4	3.7
	Chewing + Drinking		7	6.5
	Smoking + Drinking		12	11.2
	All 3 Habits		7	6.5
	None		23	21.5
	Information unavailable		4	3.7
Tumor Site	Buccal		41	38.3
	Tongue		34	31.8
	Gum		17	15.9
	FOM & palate		6	5.6
	Information unavailable		9	8.4
Tumor Size	Tis, T1 & T2		40	37.3
	T3 & T4		51	47.7
	Information unavailable		16	15.0
Lymph Node Metastasis	Negative		47	43.9
	Positive		44	41.1
	Information unavailable		16	15.0
TNM Stage	Early (I & II)		31	29.0
	Late (III & IV)		60	56.0
	Information unavailable		16	15.0
Tumor Differentiation	Well		42	39.3
	Moderate/poor		48	44.9
	Information unavailable		17	15.9
Overall survival	Range (months)	1-91	--	--
	Median	18	--	--
	Mean	22.8	--	--

### High-throughput somatic mutation detection and analysis

 The OncoCarta^™^ Panel v1.0 assay (Sequenom, San Diego, CA, USA) was used for the detection of somatic mutations because it is a rapid and cost effective method of identifying key cancer driving mutations also known as “actionable mutations” across a large number of samples. Two key advantages of using the Sequenom platform, which detects mutations based on the mass of the sequence, are 1) it has the ability to simultaneously profile multiple mutations in several genes in an large number of samples through multiplexing and 2) it can provide a 3-fold increase in mutation detection limit (as low as 5-10% of the mutant allele) compared to sequencing. In order to analyze these hotspot mutations, multiplex reactions were prepared, spotted on the SpectroChipII using the MassARRAY^®^ Nanodispenser, resolved by MALDI-TOF on the Compact Mass Spectrometer (Sequenom, San Diego, CA, USA) and analyzed using the MassARRAY^®^ Typer Analyzer software 4.0.22 where an OncoMutation™ report to indicate mutant specimens by comparing the ratios of the wild type allele peak to those of suspected mutant allele peak is automatically generated, as described by others [21,22]. The hotspot mutations that were included in this assay are tabulated in Table S3 in File S1. 

### Polymerase Chain Reaction (PCR) and direct DNA sequencing

All of the mutations that were detected by the OncoCarta^™^ Panel v1.0 assay (Sequenom, San Diego, CA, USA) were validated by direct sequencing. The *PIK3CA, BRAF, EGFR, HRAS, KRAS, NRAS* and *MET* oncogenes were also sequenced in the 16 oral cancer lines to ensure concordance between the OncoCarta^™^ Panel v1.0 assay and direct sequencing. The chosen genes were selected for their high mutation frequency in HNSCC according to the Catalogue of Somatic Mutations in Cancer (COSMIC) v60 information database (http://www.sanger.ac.uk/genetics/CGP/cosmic/) [23]. In all, 13.0% (16/123) of the total samples covering more than a third (7/19; 36.8%) of the total genes on the OncoCarta™ Panel v1.0 were sequenced for concordance between the two mutation detection methods. PCR and sequencing were performed as described previously [16,24,25]. The primers are tabulated in Table S4 in File S1. The generated sequences were compared with the reference sequences of the respective genes using the Basic Local Alignment Search Tool [26] (BLAST, NCBI, Maryland, USA; Table S4 in File S1). The frequency and spectrum of mutations were compared to those reported in COSMIC.

### Detection of TP53 somatic mutations in OSCC

The mutational status of *TP53* was determined in 112 OSCC samples that were used in the OncoCarta^™^ Panel v1.0 assay. The positive control cell lines with oncogenic mutations (n=7) and 11 OSCC samples with insufficient DNA were excluded. Mutation detection was conducted by direct sequencing of exon 4 to exon 11 where more than 85% of *TP53* mutations have been reported [27]. The procedures of PCR, purification, sequencing and analysis have been described previously [16]. The primer sequences for *TP53* are tabulated in Table S4 in File S1. The *TP53* mutations found in this study were compared to those reported in the IARC version R15 (http://www-p53.iarc.fr/) [28]. Mutations were classified into five groups: DNA binding domain (DBD), L2/L3/LSH hotspot, disruptive and truncating, and based on functional consequences, as described by others [29-31]. 

### Statistical Analysis

All statistical analyses were performed using the SPSS software (SPSS for Windows, version 16.0 (Chicago, IL) to determine statistical associations of mutations with risk habits and pathological parameters. Survival probability differences were compared by the log-rank test using Kaplan-Meier survival analysis. A *p*-value of <0.05 was considered statistically significant.

## Results

### Mutations in OSCC

 Of the 123 specimens (107 OSCC tissues, 16 OSCC cell lines), 38 (30.9%) had at least one mutation taking into account both oncogenic mutations and *TP53* mutations (Table S5 in File S1). Ten oncogenic mutations were detected in eight specimens (7 OSCC tissues and 1 OSCC cell line; 6.5%) and these mutations were found in the *PIK3CA* and *HRAS* genes. Two of the OSCC tissues had mutations in both genes (06-0005-10 and 01-002-10). The majority of oncogenic mutations were detected via the OncoCarta^™^ Panel v1.0 assay whilst others were detected via direct sequencing, as described in detail below. Of the oncogenic mutations that were identified, all but one was base transitions (Table 2). Notably, no mutations were detected in the remaining 17 oncogenes. 

**Table 2:** Oncogenic mutations in OSCC.

**Gene**	**Mutation**	**Mutation type**	**Sample**	**Mutant allele frequency**	**Site**	**pT^b^**	**pN^b^**	**pM^b^**	**Stage^b^**	**Habit**
**HRAS**	G12S	G:C > A:T	03-0004-04^a^	n/a	information unavailable	Information unavailable				BQ chewing
	G12D	G:C > A:T	01-0002-10	23%	Buccal	4	0	0	IV	BQ chewing
	G12D	G:C > A:T	06-0005-10	82%	Buccal	2	0	0	II	BQ chewing & Alcohol Drinking
**PIK3CA**	H1047R	A:T > G:C	01-0016-07	17%	Buccal	1	0	0	I	BQ chewing
	H1047R	A:T > G:C	04-0005-04	45%	Buccal	4	0	0	IV	BQ chewing & Alcohol Drinking
	E545K	G:C > A:T	01-0025-07	50%	Tongue	3	0	1	IV	None
	E545K	G:C > A:T	01-0002-10	30%	Buccal	4	0	0	IV	BQ chewing
	E542K	G:C > A:T	01-0011-10	24%	Tongue	4	1	0	IV	BQ chewing
	Q546R	A:T > G:C	150T^a^	n/a	Tongue	1	0	X	I	Alcohol Drinking
	M1043I	G:C > T:A	06-0005-10	32%	Buccal	2	0	0	II	BQ chewing & Alcohol Drinking

a Mutations were detected only through direct DNA sequencing

b Pathological characteristic

 Mutations in the *PIK3CA* gene were detected in 7/123 (5.7%) specimens. Mutations at H1047R, E545K, Q546R, E542K, and M1043I were found in six OSCC tissues and one cell line, and the mutated allele frequency ranged from 17-50% (Table 2). The Q546R mutation, not present in the OncoCarta^™^ Panel v1.0 assay, was detected in sample ORL150T by direct sequencing. *HRAS* was the only other gene that was mutated and mutations were detected in 3/123 (2.4%) of specimens. Mutations at G12S and G12D were detected in three OSCC tissues, with mutation allele frequencies of 23-82%; no mutations were detected in the cell lines (Table 2). We used seven cell lines from various tissue types as positive controls in the OncoCarta™ Panel v1.0 assay and all of the mutations that were harbored in these cell lines have been documented in Table S2 in File S1. The concordance between the OncoCarta^™^ Panel v1.0 assay and direct sequencing was 99.9% (data not shown).

Thirty three *TP53* mutations were found in 31/112 specimens (27.7%). The cell lines ORL48T and ORL195T had two *TP53* mutations respectively (Table 3). The majority of the mutations were base transitions (60.6%) with G:C to A:T being by far the most common alteration (48.5%; Table 3). Most of the mutations occurred within the DBD (81.8%), 63.6% occurred in L2/L3/LSH domain, 24.2% were hotspot mutations and 48.5% and 27.3% were disruptive and truncating mutations, respectively. Notably, the missense mutation M237K and designated hotspot mutations R175H, R248Q and R273C were found in more than one OSCC specimen (Table 3). One of the patients who had mutations in both *PIK3CA* and *HRAS*, also carried a *TP53*mutation (06-0005-10; Table 3). All except 3 samples (2.7%; ORL-115, 06-0027-05 and 11-0010-10) were negative for HPV. Two of the 3 specimens which were positive for HPV had TP53 mutations (data not shown). 

**Table 3:** *TP53* mutations in OSCC.

**Exon**	**CDS Mutation**	**Amino Acid Mutation**	**Mutation Type**	**Sample**	**Site**	**Pathological characteristic**				**Habit**	**Characterisation**				
						**pT**	**pN**	**pM**	**Stage**		**DBD**	**L2/L3/LSH**	**Hotspot**	**Disruptive**	**Truncating**
**4**	336_338delCTT	F113del	deletion	115T	Gingiva	4	x	0	IV	BQ chewing	Y	N	N	N	N
	370T>C	C124R	A:T > G:C	11-0005-07	Tongue	2	1	x	III	Smoking	Y	Y	N	N	N
**5**	454C>T	P152S	G:C > A:T	06-0051-05	Floor of Mouth	1	2	0	IV	Alcohol Drinking & Smoking	Y	N	N	N	N
	470T>G	V157G	A:T > C:G	06-0012-08	Tongue	1	0	x	I	Smoking	Y	N	N	N	N
	524G>A	R175H	G:C > A:T	01-0005-06	Gingiva	4	0	x	IV	BQ chewing	Y	Y	Y	N	N
	524G>A	R175H	G:C > A:T	166T	Tongue	2	1	0	III	none	Y	Y	Y	N	N
	527G>T	C176F	G:C > T:A	136T	Tongue	1	0	x	I	BQ chewing, Alcohol Drinking, Smoking	Y	Y	N	Y	N
	536A>G	H179R	A:T > G:C	06-0027-09	Buccal	4	2	0	IV	BQ chewing & Alcohol Drinking	Y	Y	N	N	N
	548C>G	S183*	G:C > C:G	01-0022-10	Gingiva	4	2	0	IV	none	Y	Y	N	Y	Y
**6**	614A>G	Y205C	A:T > G:C	06-0032-08	Floor of Mouth	4	2	0	IV	Alcohol Drinking & Smoking	Y	N	N	N	N
**7**	701A>G	Y234C	A:T > G:C	06-0021-09	Gingiva	4	2	0	IV	BQ chewing	Y	N	N	N	N
	702C>G	Y234*	G:C > C:G	11-0010-10	Tongue	2	0	x	II	none	Y	N	N	Y	Y
	711G>A	M237I	G:C > A:T	06-0055-10	information unavailable	4	0	x	IV	BQ chewing & Alcohol Drinking	Y	Y	N	N	N
	710T>A	M237K	T:A > A:T	06-0009-06	Buccal	2	0	0	II	BQ chewing	Y	Y	N	Y	N
	731G>A	G244D	G:C > A:T	01-0008-04	Buccal	4	2	x	IV	BQ chewing & Alcohol Drinking	Y	Y	N	Y	N
	743G>A	R248Q	G:C > A:T	04-0030-07	Floor of Mouth	4	2	x	IV	Smoking	Y	Y	Y	Y	N
	743G>A	R248Q	G:C > A:T	06-0007-04	Buccal	4	1	0	IV	BQ chewing	Y	Y	Y	Y	N
	742C>T	R248W	G:C > A:T	06-0030-10	Tongue	1	1	0	III	BQ chewing	Y	Y	N	Y	N
**8**	817C>T	R273C	G:C > A:T	04-0012-10	Buccal	2	0	0	II	none	Y	Y	Y	N	N
	817C>T	R273C	G:C > A:T	06-0019-06	Buccal	4	1	x	IV	BQ chewing, Alcohol Drinking, Smoking	Y	Y	Y	N	N
	817C>T	R273C	G:C > A:T	204T	Buccal	4	1	x	IV	BQ chewing, Alcohol Drinking, Smoking	Y	Y	Y	N	N
	831_857del24	C277_E285W	deletion	04-0014-09	Tongue	2	0	x	II	none	Y	Y	N	N	N
	832C>T	P278S	G:C > A:T	02-0004-04	Floor of Mouth	4	2	0	IV	Alcohol Drinking & Smoking	Y	Y	N	N	N
	844C>T	R282W	G:C > A:T	215T	Tongue	4	2	x	IV	Smoking	Y	Y	Y	N	N
	856G>A	E286K	G:C > A:T	06-0005-10**	Buccal	2	0	0	II	BQ chewing & Alcohol Drinking	Y	Y	N	N	N
	876delA	E294fs*51	deletion	48T	Gingiva	4	2	0	IV	none	N	N	N	Y	Y
	916C>T	R306*	G:C > A:T	207T	Tongue	1	2	0	IV	BQ chewing	N	N	N	Y	Y
**9**	960delG	K321fs*24	deletion	196T	Buccal	2	2	x	IV	BQ chewing & Alcohol Drinking	N	N	N	Y	Y
**10**	1006G>T	E336*	G:C > T:A	48T	Gingiva	4	2	0	IV	none	N	N	N	Y	Y
	1013_1014insT	F338fs*8	insertion	06-0014-08	Tongue	information unavailable				Unknown	N	N	N	Y	Y
	1024C>T	R342*	G:C > A:T	156T	Tongue	1	2	0	IV	Alcohol Drinking & Smoking	N	N	N	Y	Y

Y = Yes; N = No; * Stop codon

** Patient has 2 oncogenic mutation: G:C > A:T transition in *HRAS* gene and G:C > T:A transversion in *PIK3CA* gene

### Association of mutations with risk habits and clinico-pathological characteristics

**Figure 1:**
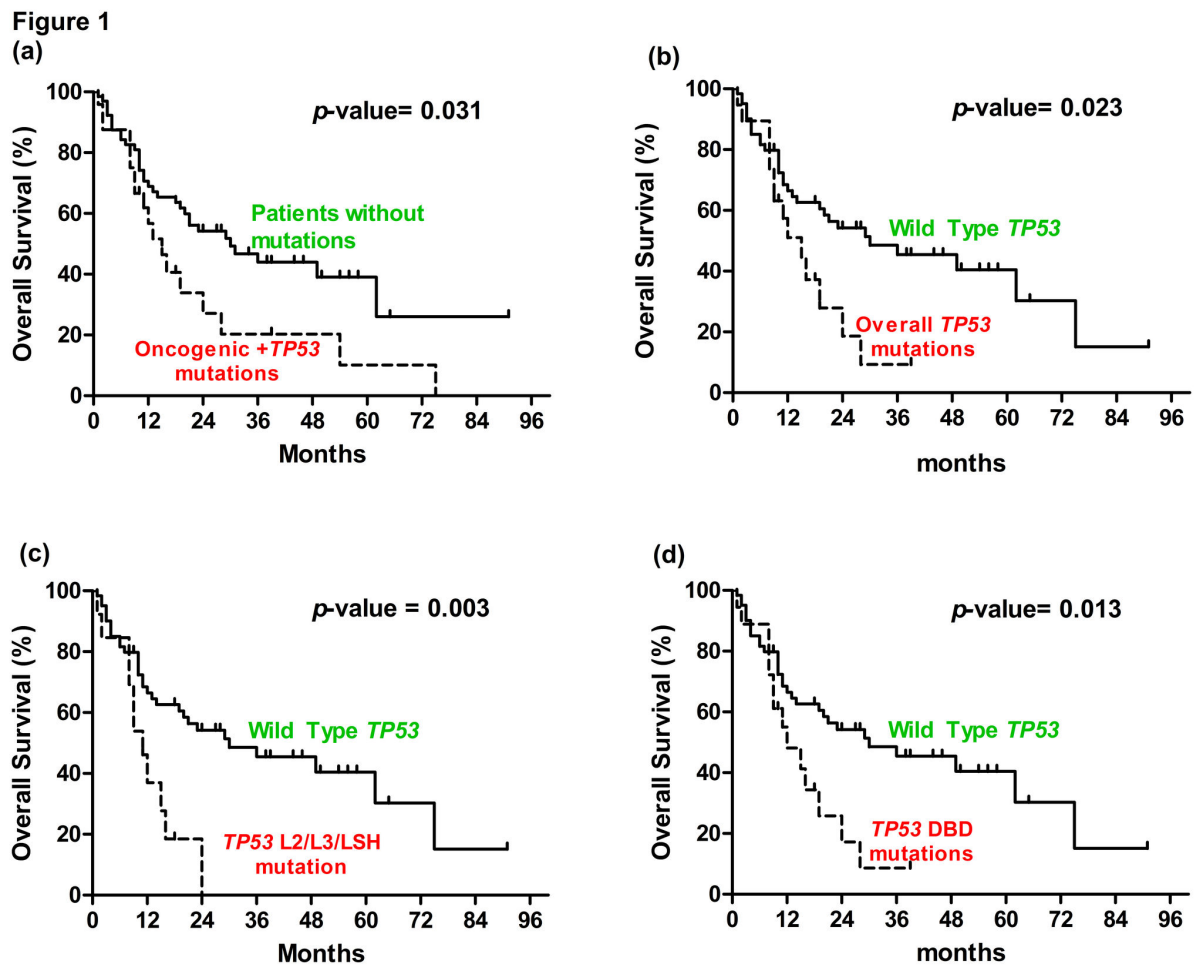
The presence of mutations in association with overall patient survival. Log Rank (Mantel-Cox) test showing that patients who harbor (a) overall *TP53* and oncogenic mutations, (b) overall *TP53* mutations, (c) L2/L3/LSH *TP53* mutations and (d) DBD *TP53* mutations have a worse overall survival compared to wild type patients.

The presence of any mutation (oncogenic or *TP53*) was not significantly associated with exposure to risk habits (Table S6 in File S1). Notably, patients with any mutation had a worse survival compared to those with a complete absence of mutations (Figure 1a). However, the presence of any mutation was not an independent factor for poor survival (Table 4). Seven out of eight OSCCs which harbored oncogenic mutations were from patients exposed to risk habits but interestingly oncogenic mutations were identified in patients who did not smoke (8/8; *p* = 0.049; Table 5). 

**Table 4:** Multivariate analysis of different types of mutations with overall survival.

**Multivariate Analysis**	***p* value**	**risk ratio (95% CI)**
**(A) Oncogenic + TP53 mutation (Wild type vs mutation)**	0.144	1.551 (0.861 - 2.794)
Age group (≤ 58 vs > 58)	0.030	1.873 (1.062 - 3.301)
Lymph Nodes Metastasis (Positive vs Negative)	<0.001	4.849 (2.102 - 11.183)
Staging (Early vs Late)	0.719	0.85 (0.350 - 2.060)
**(B) Overall *TP53* mutation (Wild type vs mutation)**	0.319	1.416 (0.715 - 2.803)
Age group (≤ 58 vs > 58)	0.037	1.906 (1.039 - 3.497)
Lymph Nodes Metastasis (Positive vs Negative)	<0.001	5.748 (2.238 - 14.76)
Staging (Early vs Late)	0.444	0.687 (0.262 -1.798)
**(C) L2/L3/LSH mutation (Wild type vs mutation)**	0.128	1.801 (0.844- 3.841)
Age group (≤ 58 vs > 58)	0.026	2.073 (1.093 - 3.930)
Lymph Nodes Metastasis (Positive vs Negative)	0.001	5.202 (2.053 - 13.183)
Staging (Early vs Late)	0.476	0.711 (0.279 - 1.815)
**(D) DNA Binding Domain mutation (Wild type vs mutation)**	0.294	1.442 (0.728 - 2.859)
Age group (≤ 58 vs > 58)	0.041	1.883 (1.026 - 3.454)
Lymph Nodes Metastasis (Positive vs Negative)	<0.001	5.628 (2.195 - 14.435)
Staging (Early vs Late)	0.429	0.68 (0.261 -1.769)

**Table 5:** Oncogenic mutations in association with risk habits and pathological characteristics.

Risk Habits/Pathological Characteristic		Patients (n)	Wildtype	oncogenic mutations	*^a^p-value*	odds ratio	95% confidence
**Overall Habit**	Any habit	94	86 (92.5%)	7 (7.5%)	0.682	2.01	(0.24-17.13)
	No habit	26	26 (96.3%)	1 (3.7%)			
**Smoking**	Ever smokers^b^	43	42 (100%)	0 (0%)	0.049	-	-
	non-smokers	77	70 (89.7%)	8 (10.3%)			
**Btel Quid chewing**	Ever chewers^b^	60	54 (90%)	6 (10%)	0.272	3.22	(0.62-16.66)
	non-chewers	60	58 (96.7%)	2 (3.3%)			
**Alcohol drinking**	Ever drinkers^b^	35	32 (91.4%)	3 (8.6%)	0.690	1.50	(0.34-6.65)
	non-drinkers	85	80 (94.1%)	5 (5.9%)			
**Lymph Node Metastasis**	Negative	54	46 (88.5%)	6 (11.5%)	0.056		
	Positive	54	54 (98.2%)	1 (1.9%)			
**TNM stage**	Early (0, I, II)	36	32 (91.4%)	3 (8.6%)	0.679		
	Late (III & IV)	72	69 (94.5%)	4 (5.5%)			

^a^Data included OSCC tissues and cell lines and analyzed by Pearson's Chi-Square Test and Fisher Exact Test

^b^Patients who ever smoke, chew, and drink may have more than one risk habit

Odds Ratio was not computed due to zero cell size

The mutational frequencies of *TP53* in patients with the different risk habits were similar (Table 6). Regardless of the nature of the risk habits, base transitions were the most common mutations (Table S7 in File S1). Truncating mutations were significantly enriched in OSCC patients with no risk habits (23.8%) compared to 4.6% in patients with at least one risk factor (*p* =0.019). All types of *TP53* mutations were enriched significantly in OSCC cell lines compared to OSCC tissues (Table 7). In addition, patients who harbored DBD and L2/L3/LSH mutations showed a worse survival probability compared to patients who had wild type *TP53* (Figure 1b, 1c, 1d) but the Cox-Regression analysis showed that *TP53* mutations were not a significant independent factor in modulating survival (Table 4). 

**Table 6:** TP53 mutations in association with risk habits and pathological characteristics.

Risk Habits/Pathological Characteristic		Patients (**n**)	Wild Type	overall *TP53* mutations	*p-value*	odds ratio	95% confidence intervals		Patients (n)	Wild Type	DBD mutations	*p-value*	odds ratio	95% confidence intervals	
**Overall Habit**	Any habit	86	62 (72.1%)	24 (27.9%)	0.605	0.774	0.293	2.044	84	62 (73.8%)	22 (26.2%)	0.823	1.135	0.372	3.465
	No habit	24	16 (66.7%)	8 (33.3%)					21	16 (76.2%)	5 (23.8%)				
**Smoking**	Ever smokers	40	29 (72.5%)	11 (27.5%)	0.781	0.885	0.374	2.096	39	29 (74.4%)	10 (25.6%)	0.989	0.994	0.402	2.460
	non-smokers	70	49 (70.0%)	21 (30.0%)					66	49 (74.2%)	17 (25.8%)				
**Betel Quid Chewing**	Ever chewers	55	39 (70.9%)	16 (29.1%)	1.000	1.000	0.439	2.277	54	39 (72.2%)	15 (27.8%)	0.619	1.250	0.519	3.012
	non-chewers	55	39 (70.9%)	16 (29.1%)					51	39 (76.5%)	12 (23.5%)				
**Alcohol drinking**	Ever drinkers	34	22(64.7%)	12 (35.3%)	0.338	1.527	0.640	3.642	32	22 (68.8%)	10 (31.2%)	0.390	1.497	0.594	3.771
	non-drinkers	76	56 (73.7%)	20 (26.3%)					73	56 (76.7%)	17 (23.3%)				
**Lymph Node Metastasis**	Negative	46	35 (76.1%)	11 (23.9%)	0.139				46	35 (76.1%)	11 (23.9%)	0.427			
	Positive	53	33 (62.3%)	20 (37.7%)					48	33 (68.8%)	15 (31.2%)				
**TNM stage**	Early (I, II)	31	20 (64.5%)	11 (35.5%)	0.617				31	20 (64.5%)	11 (35.5%)	0.288			
	Late (III & IV)	69	48 (69.6%)	21 (30.4%)					64	48 (75.0%)	16 (25.0%)				
**Risk Habits/Pathological Characteristic**		**Patients** (**n**)	**Wild Type**	**Hotspot mutations**	***p-value***	**odds ratio**	**95% confidence intervals**		**Patients (n)**	**Wild Type**	**DBD mutations**	***p-value***	**odds ratio**	**95% confidence intervals**	
**Overall Habit**	Any habit	68	62 (91.2%)	6(8.8%)	0.671	0.774	0.143	4.204	72	62 (86.1%)	3 (4.6%)	0.316	0.516	0.155	1.724
	No habit	18	16 (88.9%)	2 (11.1%)					21	16 (76.2%)	5 (23.8%)				
**Smoking**	Ever smokers	33	29 (87.9%)	4 (12.1%)	0.476	1.69	0.392	7.276	32	29 (90.6%)	3 (9.4%)	0.200	0.422	0.110	1.623
	non-smokers	53	49 (92.5%)	4 (7.5%)					61	49 (80.3%)	12 (19.7%)				
**Betel Quid Chewing**	Ever chewers	43	39 (90.7%)	4 (9.3%)	1.000	1.000	0.233	4.286	47	39 (83.0%)	8 (17.0%)	0.813	1.143	0.378	3.458
	non-chewers	43	39 (90.7%)	4 (9.3%)					46	39 (84.8%)	7 (15.2%)				
**Alcohol drinking**	Ever drinkers	24	22 (91.7%)	2 (8.3%)	1.000	0.848	0.159	4.528	26	22 (84.6%)	4 (15.4%)	1.000	0.926	0.266	3.218
	non-drinkers	62	56 (90.3%)	6 (9.7%)					67	56 (83.6%)	11 (16.4%)				
**Lymph Node Metastasis**	Negative	37	35 (94.6%)	2 (5.4%)	0.263				40	35 (87.5%)	5 (12.5%)	0.203			
	Positive	39	33 (84.6%)	6 (15.4%)					43	33 (76.7%)	10 (23.3%)				
**TNM stage**	Early (I, II)	21	20 (95.2%)	1 (4.8%)	0.432				26	20 (76.9%)	6 (23.1%)	0.540			
	Late (III & IV)	55	48 (87.3%)	7 (12.7%)					57	48 (84.2%)	9 (15.8%)				
**Risk Habits/Pathological Characteristic**		**Patients (n)**	**Wild Type**	**L2/L3/LSH mutations**	***p-value***	**odds ratio**	**95% confidence intervals**		**Patients (n)**	**Wild Type**	**Truncating mutations**	***p-value***	**odds ratio**	**95% confidence intervals**	
**Overall Habit**	Any habit	79	62 (78.5%)	17 (21.5%)	1.000	1.097	0.324	3.715	65	62 (95.4%)	3 (4.6%)	0.019	0.155	0.033	0.717
	No habit	20	16 (80.0%)	4 (20.0%)					21	16 (76.2%)	5 (23.8%)				
**Smoking**	Ever smokers	36	29 (80.6%)	7 (19.4%)	0.745	0.845	0.306	2.336	30	29 (96.7%)	1 (3.3%)	0.252	0.241	0.028	2.062
	non-smokers	63	49 (77.8%)	14 (22.2%)					56	49 (87.5%)	7 (12.5%)				
**Betel Quid Chewing**	Ever chewers	52	39 (75.0%)	13 (25.0%)	0.332	1.625	0.606	4.357	41	39 (95.1%)	2 (4.9%)	0.270	0.333	0.063	1.754
	non-chewers	47	39 (83.0%)	8 (17.0%)					45	39 (86.7%)	6 (13.3%)				
**Alcohol drinking**	Ever drinkers	30	22 (73.3%)	8 (26.7%)	0.381	1.566	0.571	4.298	24	22 (91.7%)	2 (8.3%)	1.000	0.848	0.159	4.528
	non-drinkers	69	56 (81.2%)	13 (18.8%)					62	56 (90.3%)	6 (9.7%)				
**Lymph Node Metastasis**	Negative	44	35 (79.5%)	9 (20.5%)	0.490				37	35 (94.6%)	2 (5.4%)	0.263			
	Positive	45	33 (73.3%)	12 (26.7%)					39	33 (84.6%)	6 (15.4%)				
**TNM stage**	Early (I, II)	29	20 (69.0%)	9 (31.0%)	0.251				22	20 (90.9%)	2 (9.1%)	1.000			
	Late (III & IV)	60	48 (80.0%)	12 (20.0%)					54	48 (88.9%)	6 (11.1%)				

Data included OSCC tissues and cell lines and analyzed by Pearson's Chi-Square Test and Fisher Exact Test

**Table 7:** Comparison of TP53 mutations between OSCC tissues and cell lines.

***TP53* mutation type**	**OSCC tissue samples; n=96**	**OSCC cell line samples; n=16**	p-value*
overall	21 (21.88%)	12 (75.0%)	<0.001
DBD	20 ( 20.83%)	7 (43.75%)	0.017
L2/L3/LSH	15 (15.63%)	6 (37.5%)	0.016
hotspot	5 (5.21%)	3 (18.75%)	0.032
disruptive	8 (8.33%)	8 (50.0%)	<0.001
truncating	3 (3.13%)	6 (37.5%)	<0.001

* Data were analyzed using Fisher Exact Test

## Discussion

The comprehensive profiling of cancer mutations in tumor samples has led to the detection of key perturbations that promote tumorigenesis in many types of cancers. Further, with the advent of next generation sequencing, the genomes of many types of cancers can be comprehensively characterized [32]. Such technology, however, is limited by the cost of characterizing large numbers of samples. For example, next generation sequencing data on OSCC are still limited [5-7,33] and comprehensive mutational information on OSCC amongst Asians, where the incidence is most prevalent is still unavailable. High-throughput analysis of key cancer driving mutations using mass-spectrometry remains a cost effective and efficient way of analyzing multiple mutations across a large number of samples, particularly when these are known and could influence clinical management of patients [22]. 

In this study, we examined the spectrum of oncogenic mutations across *ABL1, AKT1, AKT2, BRAF, CDK4, EGFR, ERBB2, FGFR1, FGFR3, FLT3, HRAS, JAK2, KIT, KRAS, MET, NRAS, PDGFRA, PIK3CA* and *RET* in a broad spectrum of tissues and cell lines derived from Asian OSCC. The mutation sites that were included in the OncoCarta^™^ Panel v1.0 assay are those that are frequently seen in many different types of solid tumors and are clinically actionable. Information concerning 12 of the 19 oncogenes investigated by the OncoCarta^™^ Panel v1.0 assay is either limited or absent in COSMIC for OSCC. In this study, *PIK3CA* and *HRAS* were the only two oncogenes mutated. Notably, only 6.5% of OSCC patients harbored at least one *PIK3CA* and *HRAS* mutation, whereas, these oncogenic mutations occur in 30-70% of solid tumours, including colorectal, ovarian, endometrial, lung, melanoma and breast cancer (Table S8 in File S1) [22,34]. Further, mutations in 5 of 19 genes identified by the OncoCarta^™^ Panel v1.0 assay are typically seen in many of these cancers [22,34]. With respect to lung cancer, for example, which shares similar risk factors to OSCC, mutations of *PIK3CA, HRAS, NRAS, KRAS, BRAF, EGFR, ERBB2, PDFGRA* and *RET* are seen in some 30% of patients [34]. Whole exome sequencing reported by Stransky et al. (2011) and Agrawal et al. (2011) indeed have provided us with comprehensive information on the mutation spectrum in HNSCC but their work has been confined to Caucasian samples. Interestingly, the results of the present study are similar to those reported for OSCC in patients of Caucasian origin with low mutation frequencies in *ERBB2* (1/32 patients), *FLT3* (1/38 patients) and *EGFR* (1/38 patients) [5,6]. More recently, a similar comprehensive integrative genetic analysis reported by Pickering et al. (2013) also revealed that aberrations in OSCC are commonly confined to mitogenic signaling pathway which mostly involves genes such as PI3K and RAS [7]. The results suggest that mutations within this spectrum of oncogenes appear not to be a characteristic of OSCC and, most probably, are unrelated to risk factors such as tobacco, alcohol and betel quid chewing that are historically associated with OSCC.

 Deregulation of *HRAS* is known to activate two major signaling pathways, namely, PI3K/AKT and MAPK [35,36]. In this study, only some 3% of samples contained *HRAS* mutations, findings that were surprising in view of the fact that studies in India have reported higher *HRAS* mutation frequencies [37-39] whereas those relating to Caucasian patients with OSCC range from 4-8% [5,6,40,41]. Historically, the high prevalence of *HRAS* mutations in the Indian subcontinent has been attributed to betel quid chewing [37] but the patients used in the present study were also betel quid chewers suggesting that the low mutational frequency of *HRAS* in this study was due to factors other than risk factor exposure. Other up- or down-stream proteins within the RAS pathway such as activation or over-expression of EGFR [42], and/or loss of PTEN [43] can result in the activation of the RAS signaling pathway, and may be a reason for the lack of RAS mutations in the present study. 

*PIK3CA* mutations occur frequently in many cancers including colorectal, breast, brain, gastric, ovarian and lung and 75% of these occur in exons 9 and 20 [34,44]. Hotspot mutations at these sites (E545K, E542K and H1047R) increase kinase activity and induce transformation, tumour cell proliferation, invasion and metastasis [45-47] resulting in over activated PI3K pathway as shown in *in vitro* and *in vivo* models [48,49]. Oncogenic activation of this pathway is one of the most commonly de-regulated pathway implicated in HNSCC [50]. In the present study, hotspot *PIK3CA* mutations were found in 5.7% of OSCC specimens, findings that confirm previous observations in both Asian [51,52] and Caucasian populations [5,6,9].

Importantly, the fact that oncogenic mutations occur in a small subset of OSCC patients suggests that they may benefit from targeted therapy as opposed to the conventional treatment modalities. While only a small percentage of patients may have such mutations, this translates to significant patient numbers when the global incidence of the disease is considered. *PIK3CA* mutations, for example, have been demonstrated to modulate response to mTOR- and EGFR-targeted therapy [53-55]. New generation of drugs targeting PI3K are currently being tested clinically (NCT01690871, NCT01219699, and NCT01501604) on patients with and without *PIK3CA* mutations, and results from these trials should provide further information on the role of these mutations in modulating drug response. Although the inhibition of RAS genes was relatively unsuccessful in previous studies, the activation of *HRAS* in a subset of HNSCC suggests that this could be an opportunity for the revival of drugs such as farnesyltransferase inhibitors. 

One sample in this study had both *PIK3CA* and *HRAS* activating mutations implying the significant synergistic signals of PI3K and RAS pathway critical for oral carcinogenesis may converge to activate a single downstream target that would be critical for tumorigenesis [56]. Interestingly, a recent *in vitro* study has shown that cells containing coexistence *PIK3CA* and *RAS* mutations were resistant to PI3K inhibitors [57] suggesting that coexistence of these mutations may be a predictive biomarker for resistance to PI3K inhibitors. 

In the present study, *TP53* mutations occurred in 27.7% of OSCC specimens, which is very similar to that reported in the Indian subcontinent [58,59]. It is very apparent that the *TP53* mutational frequency of OSCC patients from Asia (17-21%) [58,59] differs dramatically from those reported from the West (53-80%) [5,6,29]. The lack of *TP53* mutations in these samples were not due to involvement of HPV as only 2.7% of the samples were positive for HPV. Further, these specimens had *TP53* mutations reiterating the fact that HPV and *TP53* mutations are not mutually exclusive events in OSCC [60]. Although both *TP53* mutation and lymph node metastasis are associated with overall survival (Table 4), there was no significant association between *TP53* mutation and lymph node metastasis (Table 6). The association between *TP53* mutations and survival in the univariate analysis may reflect other functions of mutant *TP53* that is independent of metastasis. For example, mutant *TP53* have been shown to interfere with mechanisms that maintain genome integrity including DNA damage response pathways resulting in genomic instability which is a major driver of cancer development and a hallmark of cancer [61,62]. After considering other prognostic factors in the multivariate analysis, lymph node metastasis was the only significant factor associated with poor survival indicating that lymph node metastasis is a stronger driving factor in comparison to *TP53* mutations, in determining the probability of poor overall survival. Interestingly, *TP53* mutations were more prevalent in cell lines compared to OSCC tissues suggesting that they may confer an advantage during the establishment and propagation of the keratinocyte cultures. The results are consistent with previous observations where *TP53* mutations facilitate the establishment of human myeloid cell lines [63] and enhance tumor implantation *in vivo* [64]. Interestingly, the diversity of *TP53* point mutations makes this gene informative for the identification of tumor- and exposure-specific mutation patterns [65]. In the present study, 60.6% of *TP53* mutations were base transitions with G:C to A:T being the most common alteration (48.5%; Table S7 in File S1). Similarly, G:C to A:T transitions have been reported as the most predominant *TP53* mutation in OSCC in Taiwan where risk habits include the use of betel quid and tobacco [66]. However, truncating mutations in the present study were found more frequently in OSCC patients with absence of risk habits suggesting that inactivation of *TP53* may be important in the pathogenesis of OSCC. Notably, one OSCC patient in this study has three concurrent mutations in *PIK3CA*, *HRAS* and *TP53*. The prognostic significance of this remains unclear as this was only observed in one particular patient.

In summary, we show low mutation frequencies in Asian OSCC compared to a broad spectrum of solid tumours. We demonstrate that *HRAS* and *PIK3CA* mutations in Asian OSCC are uncommon but comparable to that seen in the West. *TP53* mutations, however, are significantly less common in Asian compared to Caucasian OSCC. The findings may reflect tumour heterogeneity and the diversity of risk factors between the West and India and South East Asia, but this requires verification. In the present study, the presence of actionable mutations within a few key genes may ultimately be important in clinical management. However, the data also demonstrate the urgent need for a comprehensive genetic analysis of Asian OSCC where the disease is most prevalent and where risk factors differ from those seen in the West. 

## Supporting Information

File S1 **File includes Tables S1-S8.** Table S1: Demographics and clinico-pathological characteristics of patients from which the cell lines used in this study were derived. Table S2: Positive control samples for the OncoCarta™ Panel v1.0 Assay. Table S3: Mutations in the OncoCarta™ Panel v1.0 Assay. Table S4: Primer sequences that were used for PCR and sequencing. Table S5: Mutation data across 123 samples on 19 oncogenes and *TP53*. Table S6: The presence of any mutations in relation with risk habits and pathological characterization. Table S7: Frequency of the different base changes in TP53 in patients with different risk habits. Table S8: Oncogenic mutations across common solid tumors.
(ZIP)
